# Massive Intraoperative Blood Loss Secondary to Pulmonary Artery Injury During a Video-Assisted Thorascopic Surgery: The Importance of Protocols and Preparedness

**DOI:** 10.7759/cureus.1357

**Published:** 2017-06-15

**Authors:** Brian Keyes, Erin Giles

**Affiliations:** 1 Anesthesiology, Riverside University Health System

**Keywords:** transfusion, blood transfusion, mass transfusion, surgery complications, complication

## Abstract

Hemorrhagic shock is a potentially devastating surgical condition that can present unexpectedly. This original case report involves a 57-year-old man who experienced unexpected significant blood loss as a result of iatrogenic injury to the pulmonary artery during a video-assisted thorascopic surgery. This case highlights the importance of preparedness and massive transfusion protocols in responding to intraoperative crises of this nature.

## Introduction

Video-assisted thorascopic surgery (VATS) is performed by inserting a camera and instruments into the chest via separate ports for diagnostic or treatment purposes. The potential advantages of this approach include improved wound infection rates, decreased chance of wound dehiscence, less pain, and reduced recovery times compared with open thoracotomy [1]. Significant blood loss, although rare, is a possibility due to the procedure’s proximity to large vessels. In this case, injury to the posterior descending segment of the pulmonary artery occurred during a right-sided VATS that resulted in significant, rapid blood loss leading to hemorrhagic shock and the need for massive transfusion.

## Case presentation

A 57-year-old man presented with a history of worsening fever and hemoptysis refractory to antibiotic therapy. A bronchoscopy revealed a right upper lobe necrotizing pneumonia of unknown etiology. Gram, acid-fast bacilli, and fungal stains and cultures were obtained. No significant past medical or surgical history was revealed. His social history was relevant for a 50-pack/year of smoking. His vital signs were within reference limits with the exception of an oxygen saturation of 91% on 7L oxygen by face mask. His physical examination was significant for diminished breath sounds in the right upper and lower lobes. Laboratory test results revealed a baseline hemoglobin of 9.6 g/dL and a hematocrit of 29.3%. A dense right upper lobe infiltrate with cavitation and volume loss was demonstrated on chest x-ray.

The patient granted consent for a right-sided VATS with possible lung resection and chest tube placement. The anesthetic plan included placement of a 39 French double-lumen endotracheal tube. With his recent history of hemoptysis and a hemoglobin of 9.6 g/dL, two units of cross-matched packed red blood cells were available in the operating suite. A right subclavian triple-lumen catheter and a right radial arterial line were placed prior to his arrival in the operating room. The anesthetic plan was explained to the patient and included the risks, benefits, and alternatives.

Following induction, the endotracheal tube was placed and its position was confirmed by fiberoptic visualization. The patient was positioned in the left lateral decubitus position, and the non-dependent right lung was deflated. Approximately two hours into the operation, the pulmonary vessels were identified via dissection. During the dissection, the posterior descending artery was injured during an attempt to pass a stapler across it. Until this point, the patient had a baseline tachycardia of 100 to 110 bpm and was otherwise hemodynamically stable. Significant bleeding was observed as the suction canisters rapidly filled with blood. A significant drop in blood pressure occurred instantaneously, and the patient became further tachycardic. The VATS transitioned to an open thoracotomy and right upper lobectomy. The two units of blood present in the operating room were transfused, vasopressors were administered, and the massive transfusion protocol (MTP) was initiated. Although we did have a triple-lumen central line, all three lumens were used for resuscitation; therefore, central venous pressure was not monitored. Additional peripheral intravenous lines were attempted but unsuccessful due to the limited access to the patient and his edematous state. After the two units of type and cross-matched packed red blood cells were given, we switched to type and screened blood due to the urgency. Additional blood products arrived within five minutes of initiating the MTP, and an assigned trauma nurse was present to assist with the rapid resuscitation. Per the MTP, warming measures were initiated. A total of 14 units of packed red blood cells, 12 units of fresh frozen plasma, and 12 units of platelets were transfused. The estimated blood loss was 5 L by the time we achieved hemostasis. Follow-up laboratory values, including arterial blood gasses, complete blood count, comprehensive metabolic panel, fibrinogen, and lactate, were drawn. At the time of this case, we did not have point of care coagulation studies; therefore, prothrombin time, partial thromboplastin time, and international normalized ratio were included.

Postoperatively, our patient received additional blood products and was eventually titrated off vasopressors. His acute renal injury resolved following dialysis. The patient ultimately underwent a tracheotomy and was placed on long-term antifungal medication for Aspergillus necrotizing pneumonia. The MTP facilitated the immediate availability of blood products, rapid resuscitation, and designated support staff that, in conjunction with the patient’s lack of comorbidities, contributed to the positive outcome in this case.

## Discussion

VATS has become a popular alternative to the standard thoracotomy approach as it can result in less postoperative pain and morbidity [1]. While there are benefits to this less invasive technique, it requires a great level of skill. Kawachi, et al. concluded that pulmonary vessel injury, longer operation times, and greater blood loss have frequently been observed with VATS lobectomies [2]. Massive bleeding from vascular injury is, according to Mei, et al., “considered the most troublesome and dangerous complication during VATS pulmonary resection and is an important reason for emergency conversion to thoracotomy” [3]. While catastrophic complications, such as massive blood loss, are a relatively uncommon complication of VATS, being prepared for such events is crucial for lifesaving resuscitation efforts [4].

Hypovolemic or hemorrhagic shock is a medical emergency that results from a decrease in circulatory blood volume leading to a decline in preload and cardiac output and eventually leads to the under-perfusion of tissues. In hemorrhagic shock, the rate of the volume loss is important. With a slow loss of blood, the body is often able to compensate. After significant blood loss, the compensatory mechanisms begin to fail. An acute loss is associated with higher morbidity and mortality, especially in patients with comorbidities. It is crucial that it be treated immediately before the condition leads to irreversible organ damage, including renal failure, hepatic failure, central nervous system dysfunction, and lactic acidosis [5]. Signs and symptoms of acute blood loss include hypotension, tachycardia, oliguria, and an altered mental status. A central line, arterial line, and additional large-bore venous catheters are all beneficial for monitoring and resuscitation. Vasopressors should be considered if the patient remains hypotensive despite fluid boluses. Damage control resuscitation goals must also be considered, such as combining high plasma and platelet ratios with limiting the crystalloid use and allowing permissive hypotension until hemostasis is established [6-7]. As noted in the American Society of Anesthesiologists Committee on Blood Management’s MTP for Hemorrhagic Shock, “once definitive control of bleeding has been achieved, a restrictive approach to blood product transfusion is preferred because of the well-known risks and negative outcomes of transfusion such as multiple organ failure, systemic inflammatory response syndrome, TRALI (transfusion-related acute lung injury), increased infection, and increased mortality” [8].

In this case, the cause of hypovolemic shock was iatrogenic from injury to the posterior descending segment of the pulmonary artery. The diagnosis was clear based on the patient’s clinical picture. The patient lost 5 L of blood, which approximated this patient’s total circulating blood volume. The two units of packed red blood cells already present in the operating room were not sufficient for resuscitation, which meant the MTP had to be initiated. Massive transfusion at our institution is defined as replacement approximating or exceeding the patient’s blood volume within a 24-hour interval. The MTP is initiated through a call to the hospital operator who then sends out a page to all involved teams. Six units of O-positive packed red blood cells (O-negative blood for women of childbearing age), along with six units of plasma and six units of platelets, are brought by a trauma nurse to the patient. The trauma nurse is then assigned to the patient to assist in establishing venous access and transfusion. Our hospital’s protocol includes the goal of achieving a 1:1:1 ratio of packed red blood cells, plasma, and platelets. Once an MTP is activated, the blood bank can ensure timely availability of blood products to facilitate ongoing resuscitation. Present data show that having an MTP in place results in early and more appropriate resuscitation and better outcomes compared with not having one [9], as well as improved interdepartmental communication [1].

## Conclusions

Massive blood loss is a rare but potentially catastrophic complication of VATS. The anesthesia provider and institution must be prepared to identify and treat hemorrhagic shock as a result of such complication. MTP is a valuable tool in facilitating the rapid resuscitation of patients in these situations leading to faster blood product administration, reduction in overall transfusions, correction of coagulopathy, and better patient outcomes.
